# Effect of *Cosmos caudatus* (Ulam raja) supplementation in patients with type 2 diabetes: Study protocol for a randomized controlled trial

**DOI:** 10.1186/s12906-016-1047-7

**Published:** 2016-02-27

**Authors:** Shi-Hui Cheng, Amin Ismail, Joseph Anthony, Ooi Chuan Ng, Azizah Abdul Hamid, Barakatun-Nisak Mohd Yusof

**Affiliations:** Department of Nutrition and Dietetics, Faculty of Medicine and Health Sciences, Universiti Putra Malaysia, 43400 Serdang, Selangor Malaysia; Research Centre of Excellent for Nutrition and Non-communicable Diseases, Faculty of Medicine and Health Sciences, Universiti Putra Malaysia, Serdang, 43300 Selangor Malaysia; Department of Medicine, Faculty of Medicine and Health Sciences, Universiti Putra Malaysia, Serdang, 43300 Selangor Malaysia; Department of Food Science, Faculty of Food Science and Technology, Universiti Putra Malaysia, Serdang, 43300 Selangor Malaysia

**Keywords:** *Cosmos caudatus*, Type 2 diabetes mellitus, Glycemic status, Metabolomic, Herbal medicine, Functional food, Dietary Supplementation, Randomized controlled trial

## Abstract

**Background:**

Type 2 diabetes mellitus is a major health threat worldwide. *Cosmos caudatus* is one of the medicinal plants used to treat type 2 diabetes. Therefore, this study aims to determine the effectiveness and safety of *C. caudatus* in patients with type 2 diabetes. Metabolomic approach will be carried out to compare the metabolite profiles between *C. Caudatus* treated diabetic patients and diabetic controls.

**Methods and design:**

This is a single-center, randomized, controlled, two-arm parallel design clinical trial that will be carried out in a tertiary hospital in Malaysia. In this study, 100 patients diagnosed with type 2 diabetes will be enrolled. Diabetic patients who meet the eligibility criteria will be randomly allocated to two groups, which are diabetic *C. caudatus* treated(U) group and diabetic control (C) group. Primary and secondary outcomes will be measured at baseline, 4, 8, and 12 weeks. The serum and urine metabolome of both groups will be examined using proton NMR spectroscopy.

**Discussion:**

The study will be the first randomized controlled trial to assess whether *C. caudatus* can confer beneficial effect in patients with type 2 diabetes. The results of this trial will provide clinical evidence on the effectiveness and safety of *C. caudatus* in patients with type 2 diabetes.

**Trial registration:**

ClinicalTrials.gov identifier: NCT02322268

## Background

Type 2 diabetes mellitus is one of the biggest health concerns in the world. In 2014, about 387 millions of people suffer from type 2 diabetes worldwide. The number is projected to rise to 592 million people by 2035 [1]. The prevalence of type 2 diabetes in Malaysia showed the same worrying trend. One in every five Malaysians age over 30 has type 2 diabetes [2]. Despite the drug prescription, a majority (78 %) of patients with type 2 diabetes in Malaysia still have poor glycemic control with mean HbA1C of 8.7 % [3]. Poor glycemic control is associated with an increased risk of diabetic microvascular complications including neuropathy, nephropathy and retinopathy, and macrovascular complications such as coronary heart disease [4].

Currently, the most common treatment for type 2 diabetes includes the prescription of oral anti-diabetic drugs such as metformin and sulphonylureas [5]. While the efficacy of sulphonylureas has been confirmed, their use is associated with side effects such as increased weight gain and elevated risk of hypoglycemia [6]. In addition, researchers have shown that long-term treatment with oral anti-diabetic drugs is ineffective in protecting the declining function of the pancreatic beta-cell [7, 8]. The deterioration of pancreatic beta-cell function is associated with the elevated oxidative stress in type 2 diabetic patients [9, 10]. Research indicated that oxidative damage was higher in patients with type 2 diabetes than healthy individuals [11]. Patients with type 2 diabetes had lower antioxidant capacity than healthy individuals [12]. Antioxidant potentially reduces the harmful effect of oxidative stress and consequently decreases the insulin resistance [13].

Medicinal plants have been used as traditional medicine for treating diseases, and their uses are considered safer than the oral anti-diabetic drugs [14]. The discovery of metformin, from the *Galega officinalis* has augmented the interest to quest for more natural products to treat diabetes [15]. *Cosmos caudatus*, or known locally as *Ulam Raja*, is a medicinal herb found in tropical countries [16]. It has been identified as one of the ten commonly used medicinal plants in Malaysia for treatment of type 2 diabetes [17]. *C. caudatus* contained a variety of bioactive compounds, such as ascorbic acid, quercetin, proanthocyanidin, chlorogenic acid and catechin [18–21]. *C. caudatus* has been reported to have an extremely high antioxidant capacity of about 2500 mg ascorbic acid equivalent antioxidant capacity (AEAC) per 100 g of fresh samples as compared to orange and guava which had 142 mg and 270 mg AEAC, respectively [22]. Its high antioxidant content may suggest its potential in reducing the oxidative stress [20].

In addition, *C. caudatus* have been shown to possess various medicinal benefits [16] including anti-diabetic [23], antihypertensive [24] and anti-inflammatory effect [25] in animal studies. Recent pre-clinical study in rats indicated a significantly reduction in fasting blood glucose, and a significant improvement in lipid profile after 4 weeks of supplementation [23]. Preliminary study in healthy volunteers has shown that there was a steady reduction in the postprandial blood glucose after consuming rice and fresh *C. caudatus* (unpublished data). However, the effect of *C. caudatus* in patients with type 2 diabetes remains unclear.

Metabolomics is an unbiased approach to measure metabolites disturbance within a biological system at a given time [26]. The profiling of these metabolite can provide detailed information on the metabolic pathways [27]. Additionally, metabolites levels are influenced by environmental factors such as diet and lifestyle factors [28]. Therefore, by using metabolomics approach, it provides a clearer understanding of the effect of *C. caudatus* in patients with type 2 diabetes.

The objective of this trial is to investigate the effectiveness and safety of *C. caudatus* in patients with type 2 diabetes by addressing the following hypothesis (1) *C. caudatus* supplementation will improve the glycemic status in patients with type 2 diabetes (2) *C. caudatus* supplementation will improve inflammation marker, oxidative stress marker and lipid profile in patients with type 2 diabetes (3) *C. caudatus* supplementation is safe and will not result in adverse effects (4) the therapeutic effect of *C. caudatus* will result in changes in urine and serum metabolome.

## Methods and design

### Trial design

**Fig. 1:**
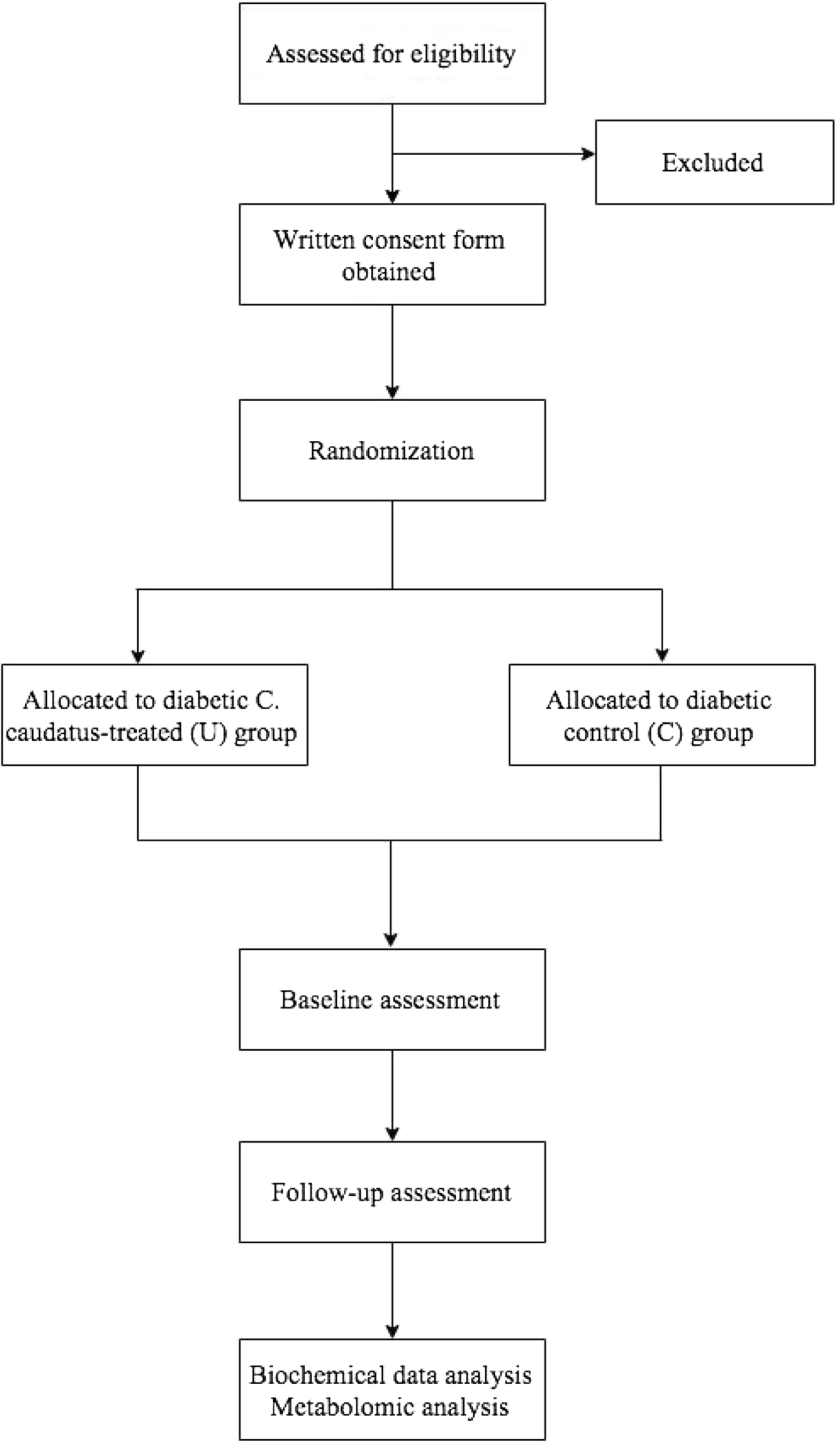
Trial flow chart

This is a single center, randomized, parallel-controlled clinical trial. A flow chart of the study protocol is shown in Fig. 1. This study will be conducted at a tertiary government hospital, Malaysia.

### Ethical approval

The study protocol has been approved by the Ethics Committee for Research involving Human Subjects Universiti Putra Malaysia (JKEUPM) (FPSK_Ogos (13)05), Herbal Medicine Research Centre, Institute for Medical Research Malaysia (version 1, 8/2014) and Medical Research and Ethics Committee Ministry of Health Malaysia (NMRR-13-1344-18177). This protocol will be conducted in compliance with Helsinki declaration.

### Randomization

Patients with type 2 diabetes will be randomly allocated to either *C. caudatus* (U) group or control (C) group. The permuted block randomization (block of 4 and 6) will be used in this study. However, researchers will be blinded to the size of each block to reduce the selection bias. All laboratory personnel will also blinded to the group allocation.

### Sample size

A total of 50 patients are required in each group to detect a significant mean difference of 1 % HbA1C [29], 1.75 % standard deviation [29], 95 % confidence level and 80 % power, with additional drop-out rate of 30 %.

### Study population

Patients with type 2 diabetes will be recruited from the outpatient medical clinic at tertiary hospital. The diagnosis of diabetes will be based on the medical record of the tertiary hospital. Eligible diabetic patients will be enrolled in the study and written informed consent will be obtained prior to recruitment at the medical clinic of the tertiary hospital.

### Inclusion criteria

Men and women between the age of 30–65 years old who have confirmed diagnosis of type 2 diabetes for more than 6 months, last HbA1C value greater than 7 %, Body Mass Index (BMI) between 18.5 and 40 kg/m^2^, treated with stabilized dose of oral anti-diabetic drugs and expected to remain on this oral anti-diabetic drugs throughout the duration of the study.

### Exclusion criteria

Patients with any of the following criteria will be excluded: 1) pregnant and lactating; 2) have any gastrointestinal disorder that interferes the bowel function, severe hepatic or renal disease (dialysis), an infection that requires antibiotics within three weeks; 3) on insulin regimen; 4) Individuals on the anticoagulant drugs such as warfarin and aspirin.

### Intervention

#### Diabetic *C. caudatus* treated (U) group

The *C. caudatus* seeds were obtained from the Institute of Bioscience, Universiti Putra Malaysia (UPM) and planted at the Universiti Putra Malaysia Agricultural Park. A voucher specimen (No. SK 2400/14) has been deposited in the herbarium, Biodiversity Unit, Institute of Bioscience, Universiti Putra Malaysia. Fresh *C. caudatus* will be provided to the *C. caudatus* treated (U) group twice a week for 8 weeks. The *C. caudatus* will be vacuum packed in a zip-lock bag before distribution. Patients in *C. caudatus* treated (U) group will be asked to consume raw *C. caudatus* leaves (15 g) daily together with one of the main meal (either lunch or dinner) for 8 weeks.

*C. caudatus* supplementation will be given simultaneously with the usual diabetes care that includes maintenance of the oral anti-diabetic drugs dosage and compliance with the dietary and physical activity recommendations. In addition, patients in *C. caudatus* treated (U) group will be advised to perform self-monitoring blood glucose. Glucometer and strips will be given to the patients to record three values daily (after fasting, before a meal and two hours after a meal). All the patients will be educated on how to use the glucometer.

#### Diabetic control (C) group

The diabetic control (C) group will be asked to abstain from consuming *C. caudatus* for 8 weeks . Both groups will be given standard lifestyle interventions which include medical nutrition therapy and physical activity enforcement). In addition, both groups will be educated for the concepts of carbohydrate exchange and serving sizes. Patients in diabetic control (C) group will be encouraged to eat the same serving of fruits and vegetable as the *C. caudatus* treated (U) group. Likewise, patients in diabetic control (C) group will be advised to perform self-monitoring blood glucose. Glucometer and strips will be given to both groups to record three values daily (after fasting, before a meal and two hours after a meal). All the patients will be educated on how to use the glucometer.

### Adherence

To check patients’ compliance during the 8 weeks, the researcher will interview them weekly by telephone. In *C. caudatus* treated (U) group, patients will be interviewed to discern whether they were consuming *C. caudatus* and to address all their concerns. In diabetic control (C) group, phone calls are made weekly to emphasize the importance of lifestyle modification.

### Patient safety

Patients will be monitored weekly during the study period and all the occurrence of the adverse events will be assessed by patient interviews. All anticipated adverse events such as loose stools, abdominal discomfort, bloating, flatulence, sign of hypoglycemia, sign of hyperglycemia will be recorded. Additionally, liver profile and renal profile will be measured at every visit.

### Study outcomes

#### Primary outcome

The primary outcome of this trial is the changes in HbA1C.

#### Secondary outcomes

Secondary outcomes in this study include changes in other glycemic parameters such as fasting glucose, insulin, fructosamine, changes in lipid profile (triglyceride, total cholesterol, HDL-cholesterol, LDL-cholesterol), changes in inflammation (high-sensitivity C-reactive protein) and oxidative stress marker (plasma malondialdehyde), changes in anthropometry parameters (BMI, waist circumference), changes in metabolite profile (urine, serum), changes in safety parameters (liver profile and renal profile), changes in blood pressure, dietary pattern and physical activity.

### Procedure

After obtaining the informed consent, both groups will be required to attend clinical research center on 4 occasions- baseline, week 4, week 8 (end of study) and week 12 (post study follow-up). Subjects will be interviewed regarding their socio-demographic background at baseline. At each follow-up, all subjects will undergo a physical examination which include anthropometric, blood pressure, blood test, urine test and completed a dietary history questionnaire and international physical activity questionnaire (IPAQ).

Anthropometry measurements including weight, height and waist circumference will be measured. Body height will be measured using SECA stadiometer model 217 (SECA, Hamburg, Germany) to the nearest 0.1 cm. Body weight will be measured using a SECA electronic scale model 703 to the nearest 0.1 kg (SECA, Hamburg, Germany). Waist circumference in cm will be assessed midway between the lowest rib and the iliac crest using a non-stretchable tape measure. BMI will be calculated using World Health Organization classification [30]. Blood pressure will be measured using an automatic blood pressure monitor (Omron, Kyoto, Japan).

Blood sample will be drawn after an overnight fasting from the antecubital vein with patients in seated position. The volume of blood taken will be 10 ml, collected in four different pre-chilled tubes. A plain evacuated blood collecting tube will be used to collect 4 ml blood for metabolomic analysis. One milliliter will be taken in potassium oxalate/sodium fluoride tubes for fasting glucose. Another 1 ml blood sample will be taken in tubes containing ethylenediaminetetraacetic acid (EDTA) for HbA1C. The remainder 4 ml blood will be collected in the heparinized tube for other biochemistry parameters. Blood will be drawn by trained nurses and immediately placed on ice in the dim light environment. Blood sample that are collected will be centrifuged at 3000 rpm for 10 min at 4 °C to obtain the serum and plasma. 15 ml urine samples will be collected in the polyethylene containers. Blood and urine sample that are collected will be stored at −80 °C until further analysis.

### Biochemical assessments

All assays will be performed using Architect ci8200 analyzer (Abbott Laboratories, USA) except glycated hemoglobin (HbA1C) and malondialdehyde. Fasting blood glucose will be performed with a hexokinase-based method (Abbott Laboratories, USA). HbA1C will be measured with turbidimetric inhibition immunoassay method (Roche Diagnostics, Germany) using Cobas Integra 800 (Roche Diagnostics, Germany). Serum total cholesterol, triglycerides, high-density lipoprotein (HDL) cholesterol, and low-density lipoprotein (LDL) cholesterol levels will be quantified by commercially available enzymatic assay kits (Abbott Diagnostics, USA). High sensitivity C-reactive protein will be determined using the quantitative immunoturbidimetric method. For renal function test, sodium, potassium and chloride will be determined by potentiometric using solid state ion-selective electrode, urea will be determined by enzymatic method and creatinine will be determined using the kinetic method. For liver function test, total protein will be determined using biuret endpoint, albumin will be determined by direct colorimetric determination, total bilirubin will be determined by diazonium salt method whereas alkaline phosphatase, aspartate aminotransferase, and alanine aminotransferase will be determined by the kinetic method. Malondialdehyde will be measured by thiobarbituric acid reactive substances assay using UV spectrophotometer (UV-1601, Shimadzu, Japan) [31]. All biochemical assays will be performed by B. P. Clinical Lab.

### Dietary intake assessment

Dietary intake will be assessed with a dietary history questionnaire. Patients will be requested to provide complete food descriptions including food and drinks (brand names), food preparation (ingredients) and cooking method (such as deep-frying, stir-frying, boiling, grilling, barbecue, roasting, and steaming) as detail as possible in a week. Pictures of food commonly consumed in Malaysia, together with a set of common household measurement tools (glass, cup, Chinese rice bowls, soup bowls, plates, teaspoon and tablespoon) will be provided to assist subjects in estimating the portion sizes of the food.

The nutrient analysis will be carried out using Nutritionist Pro^™^ diet analysis software (version 2.5, Axxya Systems, USA). This software contains several databases including Nutrient composition of Malaysian foods and the United States Department of Agriculture (USDA). For food items unavailable in Nutritionist Pro diet analysis software, database will be obtained from Singapore Food Composition Guide (Health Promotion Board, 2003) and all the nutrients for food items will be manually added to Nutritionist Pro diet analysis software.

### Physical activity levels assessment

International Physical Activity Questionnaire (IPAQ) will be administered to assess the physical activity level of the subjects. The items in the IPAQ form consist of scores on walking, moderate-intensity and vigorous-intensity activity. The IPAQ’s total score for physical activity level will be expressed as metabolic equivalents per minute (MET-min) per week, sums the duration (in minutes) and frequency of walking, moderate-intensity and vigorous-intensity activities. Levels of physical activity will be category into low, moderate and high as per IPAQ criteria [32].

### Statistical analysis

All clinical data will be performed using SPSS version 21 for windows (SPSS Inc, Chicago, USA). Data will be expressed as mean ± SD for continuous parameters, and percentage for categorical parameters. Normality tests assessed through Shapiro-Wilk tests will be carried out on each parameter before analysis. For baseline characteristic, independent t-test will be used to compare the difference in the study outcomes between the diabetic treatment and control group. All outcome measurements will be evaluated based on intention-to-treat analysis using all randomized patients who have attended the blood test at baseline. Missing data will be imputed using “last observation carried forward” method. The comparison within each group will be carried out using paired t-test, while the changes between the two groups will be conducted using independent t-test. A *p*-value of less than 0.05 will be considered as significant. Data will be reported with adherence to the CONSORT 2010 guidelines [33].

### NMR Measurements

Thawed 400 μL urine samples will be mixed with 200 μL of a 0.75 M phosphate buffer solution (containing 0.5 % sodium trimethylsilylpropionate-d4 (TSP)) prepared in D_2_O and transferred to a 5 mm NMR tube. Thawed 200 μL blood serum samples will be mixed with 400 μL D_2_O and transferred to a 5 mm NMR tube. All ^1^H NMR spectra will be collected on a Varian 500 MHz NMR spectrometer (Agilent technologies, Santa Clara, United States) equipped with a 5 mm PFG One NMR probe. One dimensional(1D) NOESY-Presaturation pulse sequence for urine and 1D Carr-Purcell Meiboom-Gill (cpmg) experiment for blood serum will be used. Each free induction decay (FID) will be collected using a total of 128 scans with a relaxation delay of 2 s.

### Multivariate data analysis for metabolomics study

All ^1^H NMR spectra will be manually phased, baseline-corrected and reference to TSP (0.0 ppm) using ACD software (ACD/Labs, Ontario, Canada). Region containing water (4.5–5.2 ppm) and TSP (0.0–0.2 ppm) will be excluded. Each ^1^H NMR spectra will be segmented in bins of 0.04 ppm. Urine spectra will be normalized based on creatinine peak and serum spectra will be normalized based on the glucose peak area. All binned data will be imported into SIMCA (Version 14.0, Umetrics, Umea, Sweden). The binned data will be pareto-scaled prior to multivariate statistical analysis. Multivariate data analysis including unsupervised principal component analysis (PCA), and supervised partial least square discriminant analysis (PLS-DA) will be performed. PCA will be used to obtain a general overview of the metabolites and to detect outliers. PLS-DA will be performed to separate different groups. The quality of the model will be described by the parameter of R^2^ and Q^2^ which represent model fitness and predictive ability. All PLS-DA models will be validated using the permutation test. An overall *p*-value of 0.05 based on the Bonferroni correction will be used.

## Discussion

Type 2 diabetes is a common chronic disease worldwide. Medicinal plants are gaining tremendous resurgence of interest among researchers recently because of their health benefits [14, 34]. *C. caudatus* is widely consumed among the local Malays in South East Asia and its potential medicinal benefits have been reported [16]. The strength of this trial is that, it is the first randomized controlled trial to determine the effectiveness of *C. caudatus* on the outcomes measurement such as glycemic status, lipid profile, oxidative stress marker, and inflammation marker which are important parameters in patients with type 2 diabetes.

To the best of knowledge, there is no established data on herb-drug interaction of *C. caudatus*. Therefore, an interim analysis will be carried out to monitor the safety of consuming *C. caudatus* throughout the study. The results of this trial will provide clinical evidence on the effectiveness and safety of *C. caudatus* supplementation in patients with type 2 diabetes. Moreover, metabolomics is a powerful tool to study the altered metabolism, identify short-term changes in biological fluids and serve as biomarker detection [26]. Metabolomics approach used in this study will fill in the gap and provide a better understanding of metabolite profile following the supplementation of *C. caudatus*. Therefore, results from this study will contribute to the knowledge on the therapeutic effect mechanism of *C. caudatus*.

To date, the most commonly used metabolomics technologies for metabolomics study are nuclear magnetic resonance (NMR) spectroscopy, and mass spectrometry (MS) such as liquid chromatography-MS (LC-MS) and gas chromatography-MS (GC-MS) [26]. GC-MS and LC-MS are robust technologies that offer high sensitivity in metabolites detection [26]. However, both of the techniques have some drawbacks including relatively slower acquisition time, requires sample derivatization (GC-MS), consumption of sample (both LC-MS and GC-MS), chromatography can drift during a sample run, which makes data processing difficult (LC-MS), and peak assignment is a major challenge (LCMS) [35, 36]. On the other hand, NMR offers some advantages such as its ability to provide high-resolution spectra, requires relatively simpler sample preparation hence more rapid analysis and more importantly non-destructive of the sample. However, NMR-based metabolomics has a lower sensitivity compared to MS [35–37].

We used NMR in this study as NMR-based metabolomics can detect many different metabolites simultaneously such as carbohydrate, amino acids, organic and fatty acids, amines and lipids. In addition, NMR-based metabolomic have shown high reproducibility and identification of the metabolites is relatively easy due to a good library of spectra [36]. The most prominent metabolites in the ^1^H NMR blood serum spectra are fat (methylene- and methyl-moieties from the lipoproteins), amino acids, lactate and glucose [38]. Meanwhile, creatinine, creatine, trimethylamide N-oxide, dimethylamide, citrate and lactate are the most significant metabolites found in the ^1^H NMR urinary spectra [39]. Recently, studies have revealed that there is a change in the amino acid metabolism in addition to the changes in glucose and fatty acid metabolism in patients with type 2 diabetes . Raised branched-chain amino acids including leucine, valine, isoleucine and glycerol were associated with the insulin assistance [40]. In the current study, NMR-based metabolomics is used to investigate the metabolic perturbation due to *C. caudatus* supplementation in patients with type 2 diabetes. To complement the current study, future studies involve MS-based metabolomics would provide further insight into the fatty-acid profiling in patients with type 2 diabetes.

However, there are limitations in this trial. Placebo is not used in this study. The reason is because there is no identical vegetable that can mimic the taste of *C. caudatus*. In addition,, there is also no blinding among the patients because they would know once they allocated in the treatment arm as they will receive the supplementation of *C. caudatus*. Therefore, the possibility of bias cannot be ruled out. In conclusion, findings from this study will provide significant evidence of using *C. caudatus* as an adjuvant therapy in patients with type 2 diabetes.
